# Signatures of alcohol use in the structure and neurochemistry of insular cortex: a correlational study

**DOI:** 10.1007/s00213-019-05228-w

**Published:** 2019-04-22

**Authors:** Sophie Betka, Lisa Harris, Charlotte Rae, Bence Palfi, Gaby Pfeifer, Henrique Sequeira, Theodora Duka, Hugo Critchley

**Affiliations:** 1 Trafford Centre, Brighton and Sussex Medical School, Clinical Imaging Science Centre, Brighton, BN1 9RY UK; 2 Behavioural and Clinical Neuroscience, School of Psychology, University of Sussex, Brighton, BN1 9QH UK; 3 University of Lille, SCALab, CNRS UMR 9193, 59045 Lille, France; 4 Radiological Science, Brighton and Sussex University Hospitals NHS Trust, Brighton, UK; 5 Sackler Centre for Consciousness Science, University of Sussex, Brighton, UK; 6 School of Psychology, University of Sussex, Brighton, UK; 7 Sussex Addiction Research and Intervention Centre (SARIC), University of Sussex, Brighton, UK

**Keywords:** Addiction, Insula, Interoception, Magnetic resonance spectroscopy, Alcohol use, Voxel/surface-based morphometry, Craving

## Abstract

**Rationale:**

Insular cortex supports the representation of motivational feelings through the integration of interoceptive information concerning bodily physiology. Compromised insular integrity is implicated in alcohol and drug use disorders. Alcohol-associated insular dysfunction may arise through aberrant glutamatergic neurotransmission associated with selective neuronal death and atrophy.

**Objective:**

In a sample of alcohol users, we combined magnetic resonance spectroscopy (MRS) with voxel and surface-based morphometry (VBM, SBM) to test the hypothesis that the neurochemical and structural properties of the insula relate to alcohol use.

**Methods:**

Twenty-three healthy individuals were characterized by measures of alcohol use and subjective craving. Right mid-insula glutamate/glutamine (Glx) and total N-acetylaspartate/N-acetyl-aspartylglutamate (TNAA) concentrations were measured using MRS. Right insular structure was quantified using VBM and SBM parameters. We tested for predictive associations between these neuroimaging and behavioral/psychometric measures using Bayesian statistics.

**Results:**

Reduced insular Glx concentration was associated with increased alcohol compulsions and, to a lesser extent, with greater alcohol use severity. Anecdotal evidence for a negative relationship between alcohol use severity and levels of insular gyrification was also observed.

**Conclusions:**

This study is, to date, the first characterization of the neurochemical and morphological integrity of insular cortex in alcohol users. Our data seem to reveal a negative relationship between alcohol use and the neurochemical and structural integrity of the insula, a critical substrate for motivational behavior. These neurobiological characteristics might contribute to loss of control toward compulsive drinking with prolonged and excessive alcohol use.

**Electronic supplementary material:**

The online version of this article (10.1007/s00213-019-05228-w) contains supplementary material, which is available to authorized users.

## Introduction

The insular cortex is implicated in the neurocircuitry of addiction: Interoceptive components of drug seeking (notably craving states) are proposed to originate within the insula (Craig 2002; Gray and Critchley 2007; Naqvi et al. 2007). Correspondingly, impairments in interoceptive bodily sensation are reported in drug-dependent users, including alcohol-dependent users (Ates Çöl et al. 2016; Sönmez et al. 2016), methamphetamine users (Stewart et al. 2014), adolescent cannabis users (Berk et al. 2015), and people with internet gaming disorder (Zhang et al. 2016). The interconnectivity between mid-insular cortex and striatal regions is implicated in the integration and expression of hedonic experience and behavior (Chikama et al. 1997; Menon and Levitin 2005; Craig 2009, 2010). Moreover, conscious access to bodily sensations has been shown to be dependent on the right insular cortex (Craig 2002, 2009, 2010; Critchley et al. 2004). Correspondingly, damage to insular cortex can change addictive behaviors (Naqvi et al. 2014). For example, insular lesions reduce nicotine craving in smokers and attenuate the occurrence of distorted cognitive appraisals that compel betting in individuals with gambling disorder (Clark et al. 2014; Abdolahi et al. 2015). Insular volume is preferentially reduced in alcohol use disorder, in the context of more diffuse gray matter shrinkage (Yang et al. 2016), and the volume and thickness of anterior insular cortex are negatively correlated to impulsivity and compulsions in alcohol-dependent individuals (Grodin et al. 2017). Major cerebral white matter tracts are also compromised in heavy drinkers, disrupting interregional connectivity in a way that predicts an enhanced functional reactivity of insular cortex to alcohol cues. Thus, structural changes may underpin exaggerated sensitivity to cues regulating alcohol consumption (Monnig et al. 2014). Individuals with alcohol dependence also show a reduction in functional interactions between insular cortex and prefrontal regions during emotional processing, indicating a generalized dysregulation of motivational and affective processes (O’Daly et al. 2012).

In alcohol use disorders, functional brain abnormalities may be attributable to changes at the neurochemical level. Alcohol interacts with glutamatergic neurotransmission, suppressing excitatory synaptic signaling, particularly through inhibition of N-methyl-D-aspartate (NMDA) receptors (Lovinger et al. 1989). This impacts synaptic plasticity by reducing long-term potentiation (Stephens et al. 2005). In compensation, the number and sensitivity of NMDA receptors increase proportionally to the amount and frequency of alcohol intake (Trujillo and Akil 1995). A sharp reduction or cessation of alcohol consumption can induce rebound neuronal hyperexcitability, leading to excitotoxicity and neuronal death (atrophy; Tsai et al. 1995). In rats, ethanol increases glutamate concentration within striatal reward circuitry (Roberto et al. 2004; Ding et al. 2012).

In humans, it is possible to quantify neurochemicals in vivo, including glutamate, using magnetic resonance spectroscopy (MRS). However, in the literature, the impact of alcohol use on glutamate concentration seems to be mixed—potentially region-specific. On one hand, in alcohol-dependent patients, glutamate concentration is increased within the left dorsolateral prefrontal cortex following detoxification and correlates with rated intensity of alcohol craving (Frye et al. 2016). In addition, increased glutamate levels are observed in the anterior cingulate cortex of patients in acute alcohol withdrawal (Hermann et al. 2012). Moreover, the combined concentration of glutamate and glutamine (Glx) correlates positively with compulsions to drink alcohol, measured by the Obsessive and Compulsive Drinking Scale (Anton et al. 1995), within cerebral structures widely interconnected with the insula such as the ventral striatum and anterior cingulate cortex (Bauer et al. 2013).

On the other hand, lower concentrations of glutamate and N-acetylaspartate (NAA) within anterior cingulate cortex are observed in the early stages of stopping drinking; these concentrations seem to normalize after 5 weeks of abstinence (Mon et al. 2012). Similarly, glutamate and NAA concentrations within anterior cingulate cortex are negatively associated with recent heavy drinking in individuals with alcohol dependence (Prisciandaro et al. 2016, 2018). Finally, lower glutamate concentration within neighboring prefrontal white matter predicts loss of control and severity of alcohol dependence in heavy drinkers (Ende et al. 2013).

Together, these observations motivate the current study, in which we tested the prediction that even the “light social” drinking of alcohol impacts the neurochemical and morphological (structural) integrity of insular cortex and related regions.

### Present study

Neurochemical imaging (MRS) studies of alcohol use disorders have focused on prefrontal or striatal reward-related areas. However, increasing evidence implicates insular cortex in specific aspects of drug seeking (notably hedonic information processing and craving states) (Chikama et al. 1997; Menon and Levitin 2005; Gray and Critchley 2007). Also, given the lateralization of the remapping of interoceptive signals at the insular level and its relationship with consciousness of such signals as well as the interconnectivity between mid-insular cortex and striatal regions (Chikama et al. 1997; Craig 2002, 2009, 2010; Menon and Levitin 2005), we focused our investigations on the right middle insular cortex. Therefore, combining MRS with behavioral and psychometric ratings, we explored the relationship between alcohol-related measures (i.e., severity, using the Alcohol Use Disorders Identification Test), craving, and compulsion (using the Obsessive Compulsive Drinking Scale) and right middle insular cortex neurochemistry (glutamate/glutamine and TNAA metabolite concentrations) in alcohol users.

We were also interested in associations between alcohol-related measures, insular neurochemistry, and insular morphology (volume and surface gyrification). Indeed, glutamatergic increases can lead to excitotoxicity which might itself lead to neuronal death and potential atrophy (Lovinger et al. 1989; Tsai et al. 1995). Furthermore, majority of studies exploring alcohol-related changes in the brain structure volume have used voxel-based morphometry (VBM). However, morphology of brain structure can also be measured using a different technique such as surface-based morphometry (SBM), which measures cortical folding and is proposed to be able to capture more subtle gray matter changes (Hutton et al. 2009; Kelly et al. 2013). For example, an extensive literature is exploring the relationship between cortical folding (i.e., gyrification) and prenatal alcohol exposure in children and adolescents (De Guio et al. 2014; Kuhn et al. 2016; Hendrickson et al. 2017. 2018). However, to our knowledge, gyrification has not been quantified in adult alcohol users. Therefore, a novel aspect of this research is the combination of MRS, VBM, and SBM to clarify the neurochemical and structural integrity of insular cortex in relation to alcohol use.

First, we expected that alcohol-related psychometric measures would all be positively related. Then, based on previous reports (Yang et al. 2016), we also predicted that insular gray matter volume and cortical gyrification index would correlate negatively with these alcohol use measures. Furthermore, based on the evidence of alcohol-induced glutamatergic excitotoxicity and due to neuronal death (Lovinger et al. 1989; Tsai et al. 1995), we hypothesized that basal insular glutamate plus glutamine concentration would be lower in individuals with higher scores on alcohol use measures, as a consequence of reduced neuronal density impacting on synaptic vesicles and glutamate-glutamine storage. Finally, we also explored if alcohol-related metabolites changes (e.g., glutamate-glutamine reduction) will be predicted by structural measures (e.g., gray matter reduction), suggesting an insular atrophy in alcohol users.

Here, MRS data were measured within a three-session within-subject experimental protocol that also tested for functional effects of a hormone, intranasal oxytocin, on interoceptive processes in alcohol users. There was no predicted effect of oxytocin administration on spectroscopic data (MRS glutamate/glutamine concentration). Evidence for a (nul) effect of oxytocin on metabolites was tested explicitly (see the “Materials and methods” section and Supplementary Information for more details).

## Material and methods

### Participants

Thirty-two male volunteers (mean age 25.1 yrs.; range 18–36 yrs) took part in the experiment. Participants were recruited via advertisements placed around the University of Sussex and Brighton and Sussex Medical School. All participants were healthy individuals. During the screening, participants were directly asked if they had any history or received any diagnosis of psychiatric or neurological diseases or if they were taking medication. Participants were also directly asked if they had any history or received any diagnosis of alcohol or drug use disorders. To be recruited, participants had to drink at least one unit (8 g) of alcohol by week. The average number of years in education was 16.9 yrs. (SD = 2.6). All participants gave written informed consent and were compensated £7 per hour for their time. The study was reviewed and approved by the BSMS Research Governance and Ethics committee.

### Procedure

The study was conducted at the Clinical Imaging Science Centre in Brighton, UK. Demographic, psychometric, and spectroscopic data were measured within a three-session within-subject experimental protocol that also tested for functional effects of a hormone, intranasal oxytocin, on interoceptive processes in alcohol users. Given the natural menstrual fluctuation of endogenous oxytocin in women, we decided to only recruit men to simplify the within-subject design procedure (e.g., women would need to be tested on the same day of their menstrual cycle). Demographic and trait psychometric data were measured during the baseline session (the first session which was not involving drug). Spectroscopic data were acquired at the end of the third (and last) session which was following the baseline session by maximum of 7 days. Each session was separated by a maximum of 2 days. Spectroscopic scan was acquired 1 h and 10 min after the drug administration. Given the short half-life of plasma oxytocin (3 to 6 min, Fabian et al. 1969), there was no predicted effect of oxytocin administration on spectroscopic data (MRS glutamate/glutamine concentration). Consistent with previous observations (Aoki et al. 2015), no evidence for an effect of oxytocin on metabolites was shown when tested for explicitly (see Supplementary Information for more details Table S1). Moreover, prior to each session, participants were breathalyzed and a urinary sample was collected to test for drug use. The urinary drug test was undertaken to confirm the absence of drug use and to exclude drug use disorder. The alcohol test was undertaken to ensure that participants abstained before the sessions. In the case of positive results, the participant would be excluded. No participants were excluded.

## Questionnaires

### Alcohol use disorders identification test

The alcohol use disorders identification test AUDIT (Babor et al. 2001) is a 10-item screening tool developed by the World Health Organization (WHO) to assess severity of alcohol use (e.g., alcohol consumption, drinking behaviors, and alcohol-related problems). Each question is scored from 0 to 4, with higher numbers indicating a greater level of risk for having or developing an alcohol use disorder.

### Obsessive-compulsive drinking scale

The Obsessive Compulsive Drinking Scale OCDS (Anton et al. 1995) is a 14-item scale measuring the obsessive and compulsive aspects of craving (e.g., drinking-related thoughts, urges to drink, and the ability to resist those thoughts and urges). The scores of “obsessions” and “compulsive drinking” subscales were calculated using published methodology (Anton et al. 1995).

### Magnetic resonance imaging and spectroscopy data acquisition

Magnetic resonance imaging (MRI) and ^1^H-MRS were performed using a 1.5 Tesla Siemens Magnetom Avanto MRI scanner with an enhanced 32-channel phased-array head coil, tuned to 63.6 MHz. A whole-brain, high-resolution T_1_-weighted 3D structural image was obtained using a magnetization-prepared gradient-echo sequence, consisting of 192 contiguous axial slices (TR = 2730 ms, Echo Time (TE) = 3.57 ms, flip angle = 7°, matrix = 256 × 256, field of view (FoV) = 256x256mm, 1.0 mm isotropic voxel size, GRAPPA acceleration factor = 2; acquisition time = 5 min 58 s). The T1-weighted image was used as an anatomical reference for each participant.

**Fig. 1:**
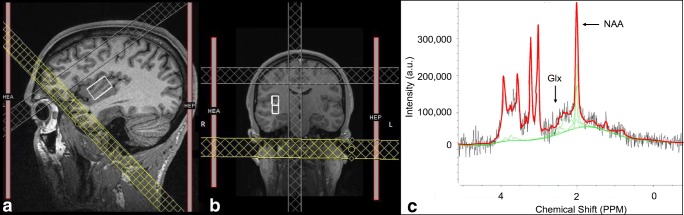
10 × 15 × 25 mm MRS voxel placement screenshot and fitted spectrum for one participant. **a** Sagittal and **b** coronal views of MRS voxel placement in the right mid-insula, and **c** example of fitted spectrum (Glx: glutamate and glutamine; NAA: N-acetylaspartate)

Using these images, a single ^1^H-MRS voxel was positioned in the right mid-insula of each participant (Fig. 1). A point-resolved spectroscopy sequence (TR = 2 s, TE = 40 ms, voxel size = 10 × 15 × 25 mm, averages = 128, flip angle = 90°; acquisition time = 4 min 24 s) was collected using a short TE of 40 ms to minimize T2 losses while removing interfering macromolecular resonances (Jang et al. 2005). A shim box of the size of the voxel was used and manual shimming was performed to minimize linewidth. An unsuppressed water sequence for use as a concentration reference was collected with four averages and other identical parameters.

### MRI and MRS data analyses

The T_1_-weighted structural MR images were segmented into gray matter (GM), white matter (WM), and cerebrospinal liquid (CSF) using Statistical Parametric Mapping 12 (SPM12; Wellcome Department of Imaging Neuroscience, University College London, UK). To calculate the tissue content of the MRS spectroscopic voxel, a binary mask of the region in each participant was created and registered to the segmented T_1_-weighted structural images. The proportion of each tissue type (tissue fractions) was calculated for the spectroscopic voxel (i.e., volume of interest) by summing the “structural” voxels of each tissue type and dividing by the total number of “structural” voxels within the volume of interest (see Supplementary Information Table S2 for details on voxel tissue content). Metabolite concentrations were then calculated and corrected (partial volume, T_1_, and T_2_ relaxation) using Gasparovic’s methods (Gasparovic et al. 2006).

Raw time-domain ^1^H-MRS data in the spectral dimension were analyzed using TARQUIN Version 4.3.5 (Wilson et al. 2011) using the unsuppressed water scan as concentration reference. The quality of the model fit was verified manually. After visual inspection, any spectrum presenting one of the following characteristics was rejected from the analyses: (1) presence of obvious spectral artifacts; (2) large baseline distortion; (3) a Cramer-Rao lower bound (CRLB) of the fit to the peak of interest greater than 20%. Based on these criteria, spectra of seven participants were discarded (four participants for large baseline distortion and CRLB > 20%; two participants for presence of obvious spectral artifacts; and one for large baseline distortion, presence of obvious spectral artifacts, and CRLB > 20%).

Metabolite concentrations in molality units of mmol/kg of tissue water were computed for total glutamate plus glutamine (Glx) and total N-acetylaspartate plus N-acetyl-aspartylglutamate (TNAA). Despite technical and methodological advances, the separation of glutamate from glutamine spectral peaks is constrained by homogeneity of the magnetic field at 1.5 Tesla. Moreover, we computed the correlation coefficient C between derived N-acetylaspartate and N-acetyl-aspartylglutamate (|C| = 0.756). Since the absolute value of the correlation coefficient between the two metabolites |C| was high (> 0.5), then the two metabolites cannot be considered sufficiently uncorrelated to permit separation (Near 2014). Thus, the sum of the two metabolite concentrations (TNAA) was reported.

### Voxel and surface-based morphometry

Voxel and surface-based morphometry (VBM, SBM) were used to identify focal differences in brain tissue composition and structure, to account for anatomical interindividual differences (Ashburner and Friston 2000; Dahnke et al. 2013). VBM and SBM were performed using the Computational Anatomy Toolbox (CAT 12 r1165, http://dbm.neuro.uni-jena.de/cat/).

T1-weighted structural images were realigned to the Anterior Commissure-Posterior Commissure. Image pre-processing followed established (default) settings in accordance with details described in the manual of CAT 12 toolbox (http://www.neuro.uni-jena.de/cat12/CAT12-Manual.pdf). In brief, T_1_-weighted 3-D images were co-registered to MNI space. Tissue probability maps derived from 452 healthy adults were used in affine registration and affine regularization, referencing ICBM space template-European brains. The affine processing parameter was set as default (“rough”). Medium strength correction for inhomogeneity was applied. Images were segmented into cerebrospinal fluid (CSF), gray matter (GM), and white matter (WM) using the “Adaptive Maximum A Posterior” technique. Mean estimates of gray matter volume (prior to normalization) were extracted for our region of interest (ROI): right insular cortex. The ROI was defined referencing the Neuromorphometrics Inc. atlas provided within CAT 12 under academic subscription (http://neuromorphometrics.com/). Gray matter volume from the anterior and posterior parts of the insular cortex was averaged to give a single composite measure of insular gray matter volume.

Global gyrification index was estimated during the tissue segmentation step (Luders et al. 2006). Local cortical gyrification index was computed within CAT12 using a high-resolution parametric mesh-based approach. This approach allows estimation of the mean curvature of the brain at different spatial scales. Large positive values (expressed in degrees) for local maxima correspond to gyri. Large negative values for local minima correspond to sulci. Values are then converted to positive values by step incorporating the absolute mean curvature. Finally, the data are smoothed using a surface-based heat kernel filter of 25 mm resulting in revealing higher values for areas with pronounced gyrification.

Mean estimates of gyrification index were extracted (prior to normalization) for our ROI, the right insula. For each participant, ROI was labeled using the Desikan-Killiany Atlas which is included in CAT 12 (Desikan et al. 2006). Analyses were undertaken to determine the extent to which any spectroscopic concentration might relate to a reduction in insular structure. Finally, the intracranial volume (TIV) was computed for each participant using the CAT12 toolbox.

### Statistical analyses

We conducted all of our analyses with the statistical software JASP, Version 0.8.6. Data were checked for potential bivariate outliers, using the function bagplot from the package “aplpack” (Rousseeuw et al. 1999) in R Version 3.5.2. Two outliers were identified and removed from the analyses. Normality of distributions was tested, given that the obsession OCDS subscale score was not normally distributed, non-parametric correlations were run.

In this present work, we tested all our hypotheses by calculating Bayesian and traditional correlation and linear regression tests. Along with frequentist statistics, we calculated the corresponding Bayes factor (BF) which was used as the basis of decision-making in respect of the compared hypotheses. A BF above 3 shows compelling evidence toward the alternative hypothesis (i.e., correlation; with 1 < BF < 3 = anecdotal evidence; with 3 < BF < 10 = moderate evidence; BF > 10 = strong evidence), whereas a BF below 1/3 provides substantial evidence toward the null hypothesis (i.e., there is no correlation; with 1/3 < BF < 1 = anecdotal evidence; with 1/10 < BF < 1/3 = moderate evidence; BF < 1/10 = strong evidence). Thus, a BF between 3 and 1/3 implies there is not enough evidence in either direction to make a firm conclusion (Jeffreys 1961; Lee and Wagenmakers 2013). Contrary to traditional statistical methods, BFs do not need to be corrected for multiple comparisons, when all evidence relevant to the theory under assay is taken into account (Gelman et al. 2012; Dienes 2016). For each test, we also reported effect size (Cohen’s f ^2; Cohen 1988).

In line with the prediction that all alcohol-related psychometric measures are positively related, we applied one-tailed non-parametric correlations (Kendall’s tau correlation coefficient; Kendall 1938) to test the putative positive relationship between the psychometric measures. Bayes factors (BF_+o_) were computed: as indicated by the subscript, the null hypothesis specified the absence of any association between the measures whereas the alternative hypothesis specified that the correlation is positive. To model the prior distribution of the alternative hypothesis, we chose the default prior of Kendall’s τ, which is a non-uniform distribution on τ produced from a uniform distribution on Pearson’s *ρ* by *parametric yoking* (van Doorn et al. 2018). The applied prior distribution of the alternative hypothesis assumes that high effect sizes are slightly less likely to be found as low effect sizes*.*

As we hypothesized that insular glutamate plus glutamine concentration would be lower in individuals with higher scores on alcohol use measures; one-tailed non-parametric correlations were again used to test the negative relationship between psychometric measures and metabolite concentrations. Bayes factors were computed (BF_-o_): as indicated by the subscript, the null hypothesis specified the absence of correlation whereas the alternative hypothesis specified that the correlation is negative. Again, we employed the default prior distribution of Kendall’s τ for the analyses (van Doorn et al. 2018). We did not correct for TIV or age as metabolite concentrations were already corrected for the VOI tissue content.

We performed Bayesian regression analyses including model selection and test of regression slopes against zero. We applied the default settings of JASP to define the prior distributions of the compared models (Rouder and Morey 2012). Linear regressions, controlling for TIV, and age, were used to test relationships between insular (ROI) volumetric/surface parameters, metabolite concentrations, and psychometric measures. The outcome and age as well as TIV, as control variables were entered in the null model. The outcome, age, and TIV, as control variables, and the predictor of interest were entered in the main model. For each model tested, we reported the BF testing for the comparison between the null and the main model. The null hypothesis was characterized by the absence of an effect of the predictor of interest on the outcome. The alternative hypothesis was characterized by the presence of an effect of the predictor of interest on the outcome. In addition to the BF, we reported effect size measures of the main model, such as R-squared and Cohen’s f ^2 (Cohen 1988). Moreover, we reported the *p* value of the model comparison procedure as well as the *p* value of the slope of interest (i.e., whether the effect of psychometric measure on the outcome is different from zero).

## Results

### Sample description

Twenty-three participants were included in the analyses. All means, standard deviations, and correlations with psychometric measures were computed (Table 1). Twenty-two percent of the sample (*n* = 5) scored between 0 and 7 on the AUDIT, suggesting absence or low level of alcohol-related problems. Thirty-nine percent of the sample (*n* = 9) scored between 8 and 15, suggesting a medium level of alcohol-related problems. Finally, 39 % of the sample (*n* = 9) scored above or equal to 16 on the AUDIT, suggesting a high level of alcohol-related problems.

**Table 1:** Means, standard deviations, one-tailed non-parametric coefficient correlations, Bayes Factors, and uncorrected *p* values for psychometric measures. For all tests, the alternative hypothesis specifies that the correlation is positive (BF _+ 0_)

		OCDS compulsion subscale	OCDS obsession subscale	AUDIT
OCDS obsession subscale	Kendall’s tau	0.401	–	
	BF_+0_	*16.00*	–	
	*p* value	*0.008*	–	
AUDIT	Kendall’s tau	0.551	0.459	–
	BF_+0_	*326.70*	*46.36*	–
	*p* value	*< 0.001*	*0.003*	–
	Mean	8.04	3.04	13.65
	SD	4.40	2.72	7.58

Support for substantial and anecdotal evidence as well as *p* value < 0.05 are represented in italics

Strong evidence for the alternative hypothesis was observed by multiple tests: AUDIT scores were positively correlated with alcohol compulsion (compulsion subscale of the OCDS; τ = − 0.551, BF_+o_ = 326.7, *p* < 0.001) and alcohol obsession (obsession subscale of the OCDS; τ = − 0.459, BF_+o_ = 46.36, *p* = 0.003, see Table 1).

### Voxel and surface-based morphometry—ROI analyses of right insula

We computed linear regression to predict structural parameters based on alcohol-related measures, while controlling for TIV and age. Means and standard deviations of gray matter volume and gyrification index, as well as, regression linear model’s R-squared, Bayes factor, Cohen’s *f* ^2, as well as uncorrected *p* values of the model and of the slope were computed (Table 2).

**Table 2:** Means and standard deviations of insular gray matter volume and gyrification index, as well as, regression linear model’s R-squared, Bayes factor, effect size, and uncorrected *p* value of the model and of the slope. For all tests, the null hypothesis was characterized by the absence of an effect of the predictor of interest on the outcome

		Gray matter volume	Gyrification index
AUDIT	*R* ^2^	0.533	0.463
	BF	0.452	*1.922*
	Cohen’s *f*^2^	0.034	*0.225*
	Model *p* value	0.002	*0.007*
	Slope *p* value	0.430	*0.052*
OCDS compulsion subscale	*R* ^2^	0.530	0.349
	BF	0.430	0.480
	Cohen’s *f*^2^	0.028	0.011
	Model *p* value	0.002	0.039
	Slope *p* value	0.480	0.651
OCDS obsession subscale	R^2^	0.525	0.386
	BF	0.398	0.726
	Cohen’s *f*^2^	0.017	0.072
	Model *p* value	0.002	0.023
	Slope *p* value	0.576	0.257
	Mean	3.78	26.04
	SD	0.31	1.12

Support for substantial and anecdotal evidence as well as *p* value < 0.05 are represented in italics

**Fig. 2:**
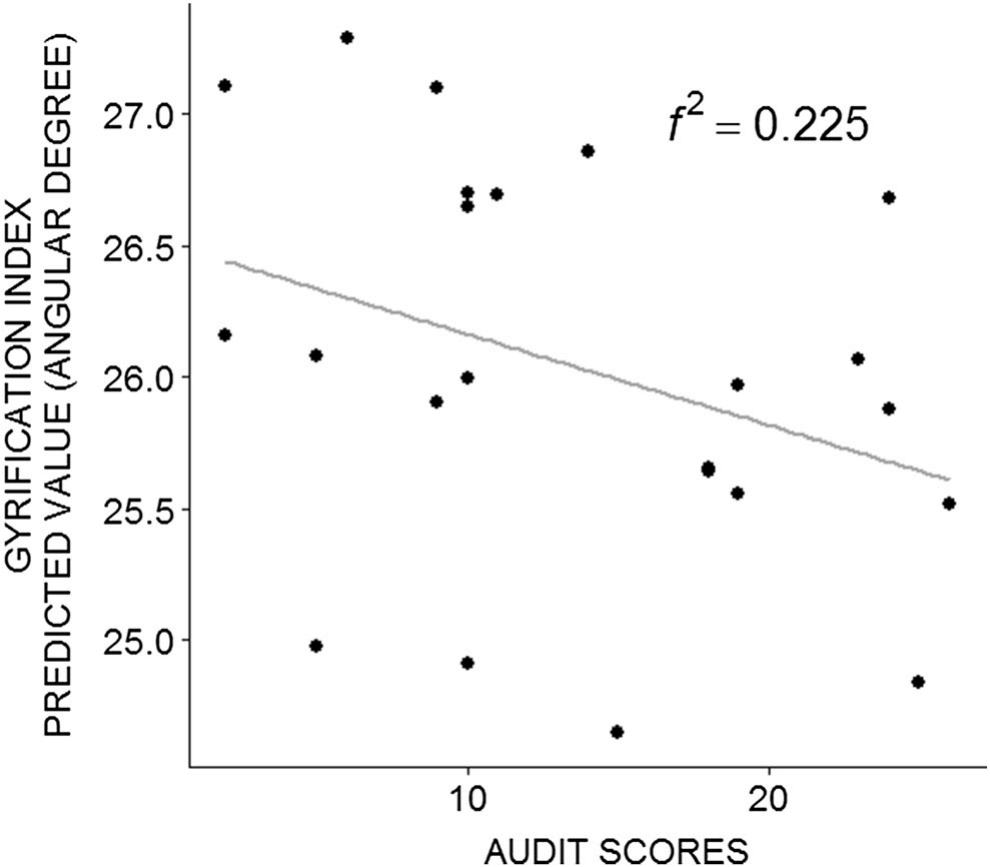
Regression between predicted surface parameters and psychometric measures. Negative relationship between right insula gyrification index and AUDIT scores (controlled for age and intracranial volume). Data were checked to exclude two outliers

Results of the linear regression predicting gyrification index from AUDIT scores, controlling for TIV, and age, showed anecdotal evidence for the alternative hypothesis: decreased gyrification index within the right insula were predicted by alcohol use severity (AUDIT) scores (Fig. 2: Model *R*^2^ = 0.463, BF = 1.922, Cohen’s *f*^2^ = 0.225; *p* = 0.007; effect of AUDIT: *β* = − 0.371, SE = 0.027, *t* = − 2.07, *p* = 0.052). The other five tests investigating the relationship between gray matter volume and psychometric measures as well as between gyrification and other psychometric measures showed anecdotal evidence for the null hypothesis (see Table 2).

### Insular magnetic resonance spectroscopy

**Fig. 3:**
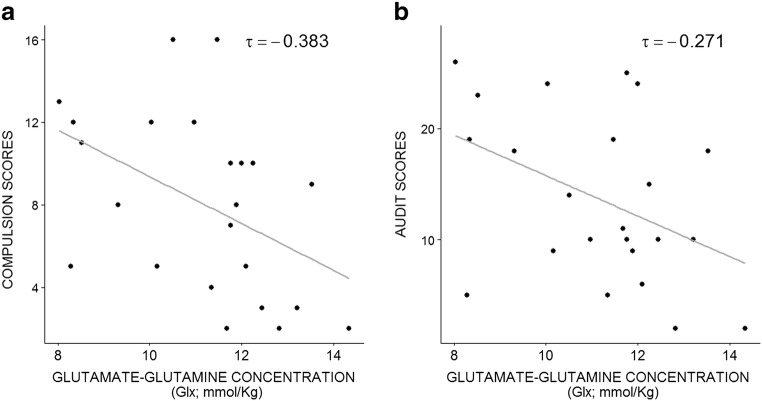
Non-parametric correlations between metabolites concentrations and psychometric measures. **a** Negative correlation between insular Glx concentration and compulsion subscale of the OCDS, and **b** negative correlation between insular Glx concentration and alcohol use severity indexed by AUDIT scores. Data were checked for potential outliers

All means, standard deviations, and correlations between metabolites and psychometric measures were computed (Table 3). Substantial evidence for the alternative hypothesis was observed: Glx concentration correlated negatively with the compulsion subscale of OCDS (Fig. 3.A: τ = − 0.383, BF_-0_ = 11.916, *p* = 0.006).

**Table 3:** Means, standard deviations, one-tailed non-parametric coefficient correlations, Bayes Factors, and uncorrected *p* values for psychometric measures. For all tests, the alternative hypothesis specifies that the correlation is negative (BF_-0_)

		OCDS compulsion subscale	OCDS obsession subscale	AUDIT	Mean	SD	
Glutamate + glutamine (Glx)	Kendall’s tau	− 0.383	− 0.270	− 0.271	11.17	1.76	
	BF_-0_	*11.916*	*2.408*	*2.453*			
	*p* value	*0.006*	0.048	0.038			
TNAA	Kendall’s tau	0.057	0.151	− 0.142	5.62	0.51	
	BF_-0_	*0.141*	*0.203*	0.674			
	*p* value	0.645	0.824	0.176			

Support for substantial and anecdotal evidence as well as *p* value < 0.05 are represented in italics

Anecdotal evidence for the alternative hypothesis was observed for the negative correlation between Glx concentration with AUDIT score (Fig. 3.B: τ = − 0.271, BF_-0_ = 2.453, *p* = 0.038) and with the obsession subscale of OCDS (τ = −0.270, BF_-0_ = 2.408, *p* = 0.048). We also observed substantial evidence for the absence of a correlation between TNAA concentration and the two subscales of the OCDS (compulsion: τ = − 0.057, BF_-0_ = 0.141, *p* = 0.645; obsession: τ = − 0.151, BF_-0_ = 0.203, *p* = 0.824). The test probing the relationship between TNAA metabolite concentration and AUDIT measures showed anecdotal evidence toward the null hypothesis (see Table 3).

### Relationship between MRS and SBM/VBM data

We tested if we could predict the Glx concentration from the gyrification index and gray matter volume while controlling for TIV and age. We observed anecdotal evidence for the alternative hypothesis (3 > BF > 1). However, more data are needed to confirm that Glx concentration relates positively to the insular gyrification index (*R*^2^ = 0.112, BF = 1.176, Cohen’s *f*^2^ = 0.12, *p* = 0.511, effect of gyrification *β* = 0.39, SE = 0.4, *t* = 1.83, *p* = 0.156). Anecdotal evidence toward the null hypothesis was observed (1/3 < BF < 1) for a non-relationship between Glx concentration and insular gray matter volume (*R*^2^ = 0.040, BF = 0.712, Cohen’s *f*^2^ = 0.03, *p* = 0.852, effect of gray matter volume *β* = − 0.25, SE = 1.823, *t* = 0.773, *p* = 0.449). However, as indicated by the Bayes factor, more data are needed to confirm or refute this finding.

## Discussion

In the present study, we tested the relationship between alcohol-related psychometric measures (alcohol use severity, alcohol compulsion), insular neurochemical (glutamate-glutamine), and structural (volume and surface gyrification) integrity by combining MRS, VBM, and SBM. First, we observed that alcohol compulsion was associated with a reliable reduction in insular glutamate-glutamine concentration. With less confidence, we observed that alcohol use severity was associated with reduced insular glutamate-glutamine concentration. Next, we found that alcohol use severity was associated with a reduction in insular gyrification. Despite their suprathreshold significance, Bayes factors for these effects indicate the need for more substantive validation.

Our initial finding was that lower combined glutamate-glutamine (Glx) concentration within the mid-insular cortex reflects an increased experience of alcohol-related compulsions; metabolites concentration reduction might underpin the compulsive facet of alcohol use. While we acknowledge the limitations of dissecting the Glx peak of nuclear magnetic resonance spectra acquired at 1.5 Tesla into glutamate and glutamine, in light of previous literature, we postulate that this effect might be explained by a meaningful association between glutamate concentration and alcohol use–related measures. The meaning of a chronic elevation/reduction of glutamate-glutamine or glutamate concentration currently remains unknown, one can nevertheless infer that something has changed in the glutamatergic system. Our data, and this postulation, extend similar published observations. Indeed, in heavy drinkers, a decrease in glutamate within frontal white matter tracts (connecting insular and cingulate cortices) predicts a subjective loss of control and shift to alcohol dependency (Ende et al. 2013). Perturbed integrity of these white matter tracts may also account for decrements in functional connectivity between insula and prefrontal/cingulate cortices, which can compromise motivational regulation in people with alcohol use disorders (O’Daly et al. 2012). Our findings are also in line with the obsessive-compulsive disorder (OCD) research literature. An MRS study noted decreased glutamate concentration in right thalamus of patients suffering from OCD: here, glutamate concentration negatively correlates with the patients’ compulsion scores (Zhu et al. 2015). While the latter study did not measure glutamate within insular cortex, nevertheless insular cortices are reciprocally connected to thalami (Mufson and Mesulam 1984; Craig 2002). Interestingly, evidence suggests that repetitive Transcranial Magnetic Stimulation (rTMS) increases glutamatergic neurotransmission (Michael et al. 2003; Yue et al. 2009; Croarkin et al. 2016) and can enhance neurogenesis in animals (Ueyama et al. 2011; Zhang et al. 2014). Such rTMS can also engender increases in cortical thickness in depressed patients (Boes et al. 2018). One could postulate that neurostimulation targeting insular cortices might attenuate alcohol compulsions, via regulation of insular glutamatergic neurotransmission and the promotion of neurotrophic insular gray matter volume recovery.

An interesting finding indicated, albeit with a need for confirmation, was that lower combined glutamate-glutamine (Glx) concentration within the mid-insular cortex reflects the severity of alcohol use. Alcohol intake and acute alcohol withdrawal are typically associated with an increased glutamatergic neurotransmission and potential excitotoxity leading to neuronal death (Lovinger et al. 1989; Tsai et al. 1995; Hwa et al. 2017). However, glutamate concentration also may depend upon individual differences in drinking patterns (Ding et al. 2012), including the consequences of binge drinking episodes. Thus, our findings may reflect the impact of recent heavy drinking within a subset of our participants. Indeed, the number of heavy drinking episodes over a fortnight correlates with decreased glutamate concentration within the anterior cingulate cortex (Prisciandaro et al. 2016, 2018; Cheng et al. 2018).

There is converging evidence for insular cortex dysfunction in drug craving and addiction (Naqvi and Bechara 2010; May et al. 2013; Migliorini et al. 2013; Dinur-Klein et al. 2014; Berk et al. 2015; Senatorov et al. 2015). However, the majority of neurochemical studies of alcohol use focus on the disruptive effect of alcohol within the ventral striatum/nucleus accumbens, where mesolimbic dopamine activity signals unpredicted reward. Despite the role of insula in normal and dysfunctional motivational experience, there is a paucity of studies that specifically test the neurochemical integrity of the insular cortex in relation to alcohol use. One study reported no neurochemical differences within insular cortex of young alcohol-dependent patients, yet an increased glutamate-to-creatine ratio within the anterior cingulate cortex (Lee et al. 2007). Another study examining how glutamate and glutamine levels relate to pain processing also measured alcohol consumption in social drinkers, but again did not find any significant associations (Zunhammer et al. 2016). Of technical importance, the majority of the previously mentioned MRS studies used creatine concentration as an external reference for glutamate concentration. However, this is likely to be problematic as the stability of creatine concentration in alcohol use disorders and recreational use is questionable (Mon et al. 2012; Tunc-Skarka et al. 2015).

Notwithstanding, our findings may also indicate a potential perturbation of the glutamate-glutamine metabolic cycle associated with alcohol use, as already suggested in alcohol-dependent patients (Thoma et al. 2011). This disruption is, putatively, a neurobiological risk factor for alcohol use disorders, rather than an incidental consequence of alcohol drinking. In this context, abnormalities in the glutamate/glutamine metabolic cycle within anterior cingulate cortex correlate with higher impulsivity in youths with a family history of alcoholism (Cohen-Gilbert et al. 2015). However, within the present study, given the low magnetic field, glutamate and glutamine concentrations are not confidently differentiable in our study. Nevertheless, our findings indicate the potential presence of alcohol-related glutamate/glutamine reduction within the right insula, which may underlie a pathogenetic vicious cycle of alcohol craving and further drinking.

Our second main findings indicate anecdotal evidence toward a negative relationship between the gyrification of the right insular cortex and alcohol drinking severity, confirming a priori predictions. To our knowledge, the present study is the first study to quantify such a relationship between insular gyrification with respect to alcohol use. Insular atrophy is commonly reported in alcohol-dependent individuals (Yang et al. 2016) and might account within our sample for part of the relationship between increased alcohol use severity and a reduction in gyrification (through gray matter loss). Notwithstanding, given the established relationship between prenatal alcohol exposure, reduced cortical folding, and higher risk to develop alcohol use disorders later in life (De Guio et al. 2014; Kuhn et al. 2016; Hendrickson et al. 2017, 2018); one cannot exclude the fact that our observations might reflect a predisposition to, rather than an effect of, alcohol consumption. Longitudinal studies are needed to disentangle this point. In addition, we observed no specific relationship between alcohol-related measures and overall gray matter volume of the insular cortex. This finding suggests that surface-based morphometry parameters might be a more sensitive measure of early alcohol-associated decline in structural gray matter organization than voxel-based morphometry estimates, as already suggested by two previous studies (Hutton et al. 2009; Kelly et al. 2013).

In our study, we also tested whether insular metabolite concentration was predicted by insular gyrification, suggesting subtle atrophy. Indeed, glutamatergic increases can lead to excitotoxicity and neuronal death (Lovinger et al. 1989; Tsai et al. 1995). It is, therefore, possible that the observed reduction in insular glutamate-glutamine concentration is a result of reduced number of cells (e.g., neural death consequent upon alcohol-related glutamatergic excitotoxicity), indexed here as subtle changes in insular gyrification. Unfortunately, our anecdotal evidence was insufficient to support this theory; future studies combining MRS and structural measures are needed to explore this point.

Nevertheless, our findings seem to indicate a combined disruption of right insular neurochemistry and structure, which seem to be associated with alcohol craving and alcohol use. The insular cortex processes afferent interoceptive signals, which are a likely basis to craving states (Critchley et al. 2004; Naqvi et al. 2007). Indeed, the disruption of interoceptive processes in alcohol-dependent individuals correlates positively with subjective craving ratings (Ates Çöl et al. 2016; Sönmez et al. 2016) and, more generally, impaired processing of bodily sensation is linked to insular cortex dysfunction in drug use disorders (Stewart et al. 2014). Recently, we observed effects compatible with an impairment in switching attention between interoceptive and exteroceptive signals in heavy drinkers (Betka et al. 2018). A similar impairment is documented in patients suffering from OCD (Stern et al. 2017). This is consistent with the important role that insular cortex plays in salience attribution (Seeley et al. 2007, Sridharan et al. 2008, Menon and Uddin 2010). Hence, reduced structural and neurochemical insular integrity might partly explain such impairment. However, it would be helpful to pursue neuroimaging studies to define functional neural correlates of pure visceral interoception (independently of other sources of bodily sensations, notably cutaneous touch or proprioceptive sensations of muscular effort) and the capacity to switch attention between salient interoception/exteroception cues, both in individuals with alcohol use disorders and those with non-clinical patterns of alcohol use.

### Limitations

The results of the present study should be considered in light of several constraints. First, we recognize that further information concerning aspects of alcohol consumption may have provided further mechanistic insight into pathoaetiological processes; including knowledge of starting age of alcohol intake, frequency, and precise quantity alcohol intake over different stages of the lifespan. Moreover, we did not formally elicit family histories of alcohol use disorder, which also impacts neurochemical ratio and morphological brain integrity (Cohen-Gilbert et al. 2015). A detailed alcohol consumption history for the preceding 2 weeks before neuroimaging may have excluded more acute factors underlying differences in neurochemical levels, as recent heavy drinking episodes can perturb glutamatergic neurotransmission (Prisciandaro et al. 2016, 2018). Also, although we controlled for alcohol and drug abstinence before the study, our findings might have been further enhanced by additionally controlling for tobacco smoking habits: Alcohol-dependent individuals who smoke show reduced N-acetylaspartate cerebral concentration when compared to alcohol-dependent non-smokers (Durazzo et al. 2013). In this regard, a strict screening for drug use or drug history should be added to protocols of future studies as many substances have long-term effects on glutamate brain level. In addition, a recognized technical limitation was the magnetic strength of the MRI scanner for neurochemical discrimination using MRS. A higher field strength can enable more direct, separate quantification of GABA, glutamate, and glutamine concentrations. Also, a greater magnetic field would increase the signal/noise ratio, which would have been beneficial given the relatively small voxel size (10 × 15 × 25 mm) necessary to derive neurochemical concentrations from insula. Bayes factors indicating anecdotal evidence for a number of effects highlight the need for a larger group size. Lastly, our sample was exclusively composed of males; therefore, further studies should verify the generalization of our findings in females.

## Conclusion

We quantified the neurochemical and morphological integrity of the insular cortex in alcohol users. Together, our data provide evidence for disruption of insular glutamate-glutamine concentration and a modulation of brain surface parameters by alcohol use. These changes may underpin a loss of control over alcohol and a shift toward compulsive drinking. Further (longitudinal) studies should explore the evolution of interoceptive processes in relation to the integrity of insular cortex through different developmental stages of alcohol and drug disorders.

## Electronic supplementary material

ESM 1 (DOCX 25 kb)

## Abbreviations

AUD: Alcohol use disorders
AUDIT: Alcohol use disorders identification test
Glx: Glutamate/glutamine
MRS: Magnetic resonance spectroscopy
NMDA: N-methyl-D-aspartate
OCDS: Obsessive compulsive drinking scale
ROI: Region of interest
SBM: Surface-based morphometry
VBM: Voxel-based morphometry
TIV: Total intracranial volume
TNAA: Total N-acetylaspartate/N-acetyl-aspartylglutamate
