# Supplementary material for: Signatures of alcohol use in the structure and neurochemistry of insular cortex: a correlational study
Source: Psychopharmacology (Berl). 2019 Apr 22;236(9):2579–91. doi: 10.1007/s00213-019-05228-w (PMC6695346; doi:10.1007/s00213-019-05228-w)
Supplement: Supplementary file 1 — (DOCX 25 kb) [file 213_2019_5228_MOESM1_ESM.docx]

**Supporting Information**

Within the current experimental protocol, oxytocin was administered to half the participants in a manipulation that addressed a different experimental hypothesis. We tested if there was an unlikely influence of this transient manipulation (40 IU of oxytocin nasal spray - Syntocinon Novartis, Basel, Switzerland- or placebo - same composition as Syntocinon except for oxytocin) on metabolite concentration. Spectroscopy data were acquired 1 hr and 20 min post-inhalation.

We computed the Bayes factors using the default prior (two-tailed Cauchy distribution with a scale of 0.707 on the standardised effect size) defined by Rouder and his colleagues (2009). Analyses were done using JASP, Version 0.8.6 and in IBM SPSS, version 24. The alternative hypothesis specified the presence of an effect of the drug on metabolite concentration. Independent samples t-tests showed no evidence for the effect of oxytocin on metabolite concentrations (Table S1). Specifically, neither the Bayes factor in favour of the effect of oxytocin on glutamate-plus-glutamine nor the Bayes factor favouring the effect of oxytocin on TNAA passed the level of BF = 3 (Glx: BF= 0.459; *p* =0.45; TNAA: BF= 1.053; *p* =0.107).

Rouder, J. N., Speckman, P. L., Sun, D., Morey, R. D., & Iverson, G. (2009). Bayesian t tests for accepting and rejecting the null hypothesis. *Psychonomic bulletin & review*, *16*(2), 225-237.

**Tables**

Table S1

|  | DRUG | N | Mean | Std. Dev | Std. Error Mean | T-test (P value) | Bayes  Factor |
| --- | --- | --- | --- | --- | --- | --- | --- |
| GLUTAMATE + GLUTAMINE (Glx) | PLACEBO | 12 | 11.54 | 2.70 | 0.78 | 0.450 | 0.459 |
|  | OXYTOCIN | 13 | 10.77 | 2.28 | 0.63 |  |  |
| TNAA | PLACEBO | 12 | 5.42 | 0.57 | 0.16 | 0.107 | 1.053 |
|  | OXYTOCIN | 13 | 5.75 | 0.39 | 0.11 |  |  |

Table S1: Independent samples t-test, *p* values and Bayes factors between oxytocin and placebo pools of participants.

Table S2

| **Subjects** | **fGM** | **fWM** | **fCSF** |
| --- | --- | --- | --- |
| 1 | 0.71 | 0.04 | 0.25 |
| 2 | 0.83 | 0.05 | 0.12 |
| 3 | 0.85 | 0.08 | 0.07 |
| 4 | 0.72 | 0.02 | 0.26 |
| 5 | 0.84 | 0.02 | 0.13 |
| 6 | 0.59 | 0.02 | 0.39 |
| 7 | 0.78 | 0.02 | 0.2 |
| 8 | 0.81 | 0.07 | 0.12 |
| 9 | 0.82 | 0.03 | 0.15 |
| 10 | 0.68 | 0.01 | 0.32 |
| 11 | 0.79 | 0.13 | 0.08 |
| 12 | 0.83 | 0.07 | 0.1 |
| 13 | 0.8 | 0.02 | 0.18 |
| 14 | 0.62 | 0.02 | 0.36 |
| 15 | 0.68 | 0.02 | 0.31 |
| 16 | 0.79 | 0.02 | 0.19 |
| 17 | 0.67 | 0.03 | 0.29 |
| 18 | 0.72 | 0.02 | 0.26 |
| 19 | 0.8 | 0.04 | 0.16 |
| 20 | 0.72 | 0.02 | 0.26 |
| 21 | 0.79 | 0.02 | 0.19 |
| 22 | 0.75 | 0.09 | 0.16 |
| 23 | 0.73 | 0.06 | 0.21 |
| 24 | 0.72 | 0.02 | 0.26 |
| 25 | 0.72 | 0.01 | 0.27 |

Table S2: Tissue content of the MRS spectroscopic voxel for each participant (fGM = fraction of grey matter; fWM = fraction of white matter; fCSF = fraction of cerebrospinal fluid).
